# Prognosis of prostate cancer and bone metastasis pattern of patients: a SEER-based study and a local hospital based study from China

**DOI:** 10.1038/s41598-020-64073-6

**Published:** 2020-06-04

**Authors:** Dongyu Liu, Yue Kuai, Ruohui Zhu, Chenhe Zhou, Yiqing Tao, Weidong Han, Qixin Chen

**Affiliations:** 1 Department of Orthopedics Surgery, 2nd Affiliated Hospital, School of Medicine, Zhejiang University, Hangzhou, China; 2 Department of Medical Oncology, Sir Run Run Shaw Hospital, College of Medicine, Zhejiang University, Hangzhou, Zhejiang China; 3 Department of Internal Medicine, Lincoln Medical Center,234 E149th Street, The Bronx, NY 10451 USA

**Keywords:** Cancer epidemiology, Urological cancer

## Abstract

Prostate cancer (PCa) is the leading cause of cancer-related death among men worldwide. Knowledge of the prognostic factors of PCa and the bone metastasis pattern of patients would be helpful for patients and doctors. The data of 177,255 patients with prostate cancer diagnosed between 2010 and 2013 with at least five years of follow-up were retrieved from the Surveillance, Epidemiology, and End Results (SEER) database. Multivariate Cox regression analysis was used to determine the predictive value of patients’ characteristics for survival after adjusting for other variates. Multivariate logistic regression analysis was used to evaluate the odds ratio of bone metastasis in PCa patients. The predictive value of age, race, marital status, and tumor characteristics were compared. The survival of patients with different socioeconomic statuses and bone metastasis statuses was compared by Kaplan–Meier analysis. A total of 1,335 patients with prostate cancer diagnosed between 2009 and 2015 were enrolled from the Second Affiliated Hospital of Zhejiang University School of Medicine. The survival of patients with different prostate-specific antigen (PSA) levels, Gleason scores, marital statuses and bone metastasis statuses was compared by Kaplan-Meier analysis. In SEER database, 96.74% of patients were 50 years of age or older. Multivariate Cox analysis revealed that for PCa patients, age at presentation, older age, single marital status, lower socioeconomic status, higher PSA level, T1 and N0 stage, and bone metastasis were independent risk factors for increased mortality. Multivariate logistic regression analysis revealed that patients who were married, were living in urban areas, had lower PSA levels, underwent surgery, and radiation had lower OR factors for bone metastasis. Asian or Pacific Islander, better socioeconomic status, lived in urban areas, married marital status, lower PSA levels and lower Gleason scores were better prognostic factors in PCa. Additionally, patients with single or divorced marital status, who were living in rural places had higher PSA levels, and T1 and N0 stages have a high OR for bone metastasis.

## Introduction

Prostate cancer (PCa) is one of the top ten leading causes of cancer-related death among men in China, the second in the United States, and the third in Europe^1–3^. Previous studies showed that advanced age is the primary risk factor, with more than 75% of all prostate cancers diagnosed in men aged 65 years old^4^. Geographic variation may be attributed to the different incidence rates around the world. For most men, prostate cancer is slow-growing, does not lead to clinical signs in the early stage and is usually detected by routine testing^5^. However, in some men, PCa progresses quickly and can cause hematuria or urinary obstruction. Cancer that spreads past the gland may result in lower extremity edema from regional lymphatic obstruction or pain from bone metastasis.

Patients’ social and clinical characteristics would have important effect on prognosis. Social characteristics including kind of race, marital statues, living in rural or urban places, education level, family income, and percentage of poor people in city. Some research reported that married individuals would enjoy lower mortality and longer overall survival compared to those who were single, separated, widowed, or divorced persons^6–9^. Men living in urban areas were likely to receive definitive treatment for their early-stage prostate cancer than those who living in rural areas^10^. Education level and neighborhood socio-economic status were independently associated with risk of advanced PCa^11^.

Patients clinical characteristics including prostate-specific antigen (PSA) level, Gleason scores, histological grade, clinical stage, TNM stage, treatment therapy and metastasis status. PSA testing and digital rectal examination (DRE) are used as primary screening tools in the early detection of prostate cancer. Trans-rectal ultrasound (TRUS) and TRUS-guided needle biopsies are performed to confirm diagnosis following PSA or DRE testing^12^. Effective early detection and treatment strategies in asymptomatic men would potentially provide a great benefit.

Unfortunately, some patients suffer from advanced PCa within two years due to the development of bone metastases^13,14^. Approximately 10% of newly diagnosed PCa patients present with bone metastasis, increasing to 80% at advanced stages of the disease^15,16^. Butler SS *et al*. reported that the incidence of localized prostate cancer declined across age groups from 2012 to 2015, however, the incidence of distant metastatic disease increased gradually^17^. These metastases are associated with poor prognosis, bone pain, and pathological characteristics and indicate the incurability of disease in most cases^18^.

In the current study, we aimed to identify risk factors associated with cancer prognosis and to detect the bone metastasis pattern of PCa patients in SEER database and a Chinese hospital. We compared the consistency and difference between SEER data and local hospital data. We were particularly interested in isolated metastasis to the bone, as bone metastasis has a worse outcome and prognosis in PCa. The identification of the prognostic factors and pattern of bone metastasis in PCa may guide clinicians, researchers and patients to better understand this cancer.

## Methods

### SEER study population

Data were obtained from the Surveillance, Epidemiology, and End Results (SEER) database, which covers approximately 30% of the population in the US, by SEER*Stat, version 8.3. The current data were based on 18 registries in the SEER database released in November 2018^19^. In the present study, we identified a cohort of patients who were aged ≥18 years with a histological diagnosis of PCa between 2010 and 2013 and at least five years of follow-up. Survival time was defined as the time from diagnosis to the date of death, censored at the last follow-up or alive until the last follow-up. According to the International Classification of Diseases for Oncology, third edition (ICD-O-3), prostate cancers were identified by tumor site code C61.9^11^. An additional criterion was used to to further defined the study population: one primary cancer only. This study was exempt from institutional review board review due to its public nature and the identification of all data

### Local patient population

A total of 1,335 patients were enrolled from the Second Affiliated Hospital, Zhejiang University. In the present study, we identified a cohort of patients who were aged ≥18 years with a pathological diagnosis of PCa only between 2009 and 2015 and at least four years of follow-up. Survival time was defined as the time from diagnosis to the date of death, censored at the last follow-up or alive until the last follow-up. The date collected from the Second Affiliated Hospital of Zhejiang University School of Medicine was approved by the Ethics Committee of Zhejiang University (Hangzhou, China).

### Patient characteristics

Patient characteristics were extracted from the SEER database. Age, sex, race, marital status, rural or urban places, social status, and tumor characteristics were included. The patients were categorized into two age groups (18–49 years old, and ≥50 years old) in the chi-square test. Race was classified as white, black, Asian or Pacific Islander or others. Marital status included “single,” “married,” and “divorced, widow, separated, or domestic partner.” Rural and urban places were categorized according the rural-urban codes 2013. We also extracted the socioeconomic status (SES) of each patient, which is based on the county of residence. The county-level socioeconomic measures of interest included median family income, percentage of persons living below the poverty line, and percentage of persons at least 25 years of age with less than a high school education. These county-level data were used to create a composite SES^20–23^. In briefly, the percentage of persons at least 25 years of age with less than a high school education, median family income, and percentage of persons living below the poverty line were each divided into quartiles. All three socioeconomic variables were equally weighted and added together to create the composite SES score. In other words, a higher score means a higher income, less poverty, and more education. The score ranged from 3 to 12 (low, SES score ≤3; middle, SES score 4–10; high, SES score ≥11). However, because of the lack of data on socioeconomic status in local hospitals, we did not analyze the SES status of local patients.

The tumor characteristics included PSA level, Gleason Score, histologic grade, tumor stage, T stage, N stage, treatment condition, and bone metastasis. Histologic grade was categorized into well-differentiated, moderately differentiated, poorly differentiated, or undifferentiated. Cases with variable values other than those specified (including unknown values) were excluded from the final analysis set.

### Statistical considerations

We compared characteristics of patients with aged 18–49 and ≥50 by the chi-square tests. Then, we calculated the overall one-year and five-year survival rates of prostate cancer patients by Kaplan–Meier(KM) curves, and survival differences were examined by the log-rank test. We used a multivariable Cox model to estimate hazard ratio (HR) of cancer according to the survival outcomes of prostate cancer patients. Finally, we used multivariable logistic regression model to estimate odds ratio (OR) of bone metastasis of prostate cancer patients. *P* < 0.05 was considered statistically significant, and all analyses were performed by STATA, version 12.0 (Stata Corp, College Station, TX, USA) and GraphPad Prism (Graphpad 6.0).

### Ethics approval and consent to participate

As the data used was extracted from SEER dataset (public), Ethics approval and Consent to participate could be checked in SEER. We were permitted to have Internet access after our signed data-use agreement (http://seer.cancer.gov/data/sample-dua.html) was approved by the SEER administration (Reference number: 12949-Nov2018). The date collected from the Second Affiliated Hospital of Zhejiang University School of Medicine was approved by the Ethics Committee of Zhejiang University (Hangzhou, China).

## Results

### Patient demographics

We identified 177,255 individuals with a diagnosis of prostate cancer, from January 1, 2010, to December 31, 2013. The characteristics shown in Table S1 include the clinical characteristics and tumor features of patients with prostate cancer. In this population, 3.26% of patients were under the age of 50, 96.74% of patients were 50 years of age or older (Table 1). Most of them were white, married, and live in urban places. Approximately 70% of patients had middle socioeconomic status, 20% had high socioeconomic status, and 10% had low socioeconomic status. The PSA level in these two age groups was slightly different. The PSA level of “under 10 ng/mL” in the under 50 group was 73.73%, while that in older than 50 group was 63.10%. There were more patients in the older group with PSA level of 10–20 ng/mL (12.78% vs. 8.30%). Most of patients had a Gleason score under 6. There were more patients with a poorly differentiated grades in the older group than in the younger group (54.85% and 45.60%, respectively). More patients in the younger group underwent surgery (63.66%) compared with those in the older group (39.83%). However, the number of patients who did not have surgery recommended in the older group was greater than that in the younger group (53.71% and 31.39%, respectively). Interestingly, the percentage of patients in each group with bone metastasis was roughly the same (2.97% and 3.79%).

**Table 1:** Crude and adjusted probability of prostate cancer specific and all cause mortality. Hazard Ratios (HR) are adjusted for age, race, marital status, PSA, Gleason score, Stage, AJCC staging (TNM), therapy, bone metastasis. The model for prostate cancer specific mortality treats non-prostate cancer deaths as censored observation.

Character	Prostate Cancer Specific Mortality	*P* value	All-Cause Mortality	*P* value
	HR (95%CI)		HR (95%CI)	
**Age**				
<50	ref.		ref.	
50-59	1.045 (0.835-1.307)	0.698	1.241 (1.046-1.473)	0.013
60-69	1.159 (0.932-1.441)	0.186	1.680 (1.422-1.983)	<0.001
70-79	1.737 (1.393-2.165)	<0.001	3.028 (2.561-3.579)	<0.001
≥80	3.200 (2.551-4.003)	<0. 001	6.571 (5.539-7.800)	<0.001
**Race**				
White	ref.		ref.	
Black	1.013 (0.935-1.098)	0.703	1.193 (1.136-1.253)	<0.001
Asian or Pacific Islander	0.671 (0.574-0.785)	<0.001	0.682 (0.616-0.756)	<0.001
**Marital status**				
Single	ref.		ref.	
Married	0.767 (0.702-0.837)	<0.001	0.662 (0.626-0.700)	<0.001
Divorced/seprated/widowed/domestic partner	0.999 (0.902-1.105)	0.967	0.982 (0.921-1.047)	0.576
**Rural-urban**				
Urban	ref.		ref.	
Rural	1.036 (0.958-1.120)	0.470	1.164 (1.110-1.222)	<0.001
**Composite SES**^a^				
≤3	ref.		ref.	
4–10	0.857 (0.781-0.940)	0.001	0.823 (0.778-0.872)	<0.001
≥11	0.731 (0.650-0.821)	<0.001	0.706 (0.657-0.760)	<0.001
**PSA** (**ng/mL)**				
<10	ref.		ref.	
10–20	1.890 (1.711-2.089)	<0.001	1.525 (1.446-1.609)	<0.001
>20	3.100 (2.818-3.409)	<0.001	2.137 (2.019-2.261)	<0.001
**Gleason Score**				
≤6	ref.		ref.	
7	0.284 (0.071-1.141)	0.077	0.992 (0.646-1.525)	0.972
≥8	2.820 (1.554-5.115)	0.001	2.131 (1.282-3.543)	0.004
**Grade**^b^				
G1	ref.		ref.	
G2	0.887 (0.486-1.620)	0.697	0.941 (0.756-1.172)	0.582
G3	2.885 (1.593-5.226)	<0.001	1.500 (1.206-1.864)	<0.001
G4	6.470 (3.344-12.517)	<0.001	2.308 (1.638-3.252)	<0.001
**Stage**				
I	ref.		ref.	
II	0.272 (0.035-2.113)	0.213	0.321 (0.155-0.665)	0.002
III	0.501 (0.064-3.909)	0.509	0.382 (0.182-0.799)	0.011
IV	1.65 (0.701-12.914)	0.629	0.745 (0.358-1.554)	0.433
**T stage**				
T1	ref.		ref.	
T2	0.889 (0.825-0.958)	0.002	0.891 (0.851-0.933)	<0.001
T3	0.798 (0.708-0.900)	<0.001	0.779 (0.702-0.865)	<0.001
T4	1.504 (1.341-1.686)	<0.001	1.522 (1.378-1.681)	<0.001
**N stage**				
N0	ref.		ref.	
N1	1.236 (1.133-1.348)	<0.001	1.277 (1.180-1.381)	<0.001
**Surgery**				
Yes	ref.		ref.	
Not recommendation	1.779 (1.623-1.950)	<0. 001	1.886 (1.778-2.000)	<0.001
Refused	1.814 (1.550-2.123)	<0.001	1.802 (1.639-1.978)	<0.001
**Radiotherapy**				
Yes	ref.		ref.	
No	1.174 (1.092-1.262)	<0.001	1.236 (1.182-1.293)	<0.001
**Bone metastasis**				
Yes	ref.		ref.	
No	0.341 (0.306-0.381)	<0.001	0.404 (0.369-0.444)	<0.001

^a^Composite socioeconomic status.

^b^G1: well differentiated; G2: moderately differentiated; G3: poorly differentiated; G4: undifferentiated; NOS: not otherwise specified; SEER: Surveillance, Epidemiology, and End Results.

### SEER survival analysis

Multivariable competing risk regression models tested the effect of patient characteristics including age, race, marital status, place of living, socioeconomic status, PSA level, Gleason score, and tumor characteristics on cancer-specific mortality (CSM) and all-cause mortality (ACM) in 177,255 prostate cancer patients (Table 1).

Age is an independent prognostic factor. In the ACM model, we found that older patients had higher mortality. While in the CSM model, patients older than 70 years old have a poor prognosis compared with those who are younger.

Asian and Pacific Islander patients had a better prognosis with white patients as a reference, the patients who were Asian or Pacific Islander have a better prognosis in the CSM and ACM models (HR, 0.671 95%CI 0.574–0.785, *P* < 0.001; and HR 0.682, 95%CI 0.616–0.756, *P* < 0.001, respectively). In the ACM model compared with white patients, patients whose race was “Black” have a worse outcome (HR 1.193, 95%CI 1.136–1.253, *P* < 0.001).

**Figure 1:**
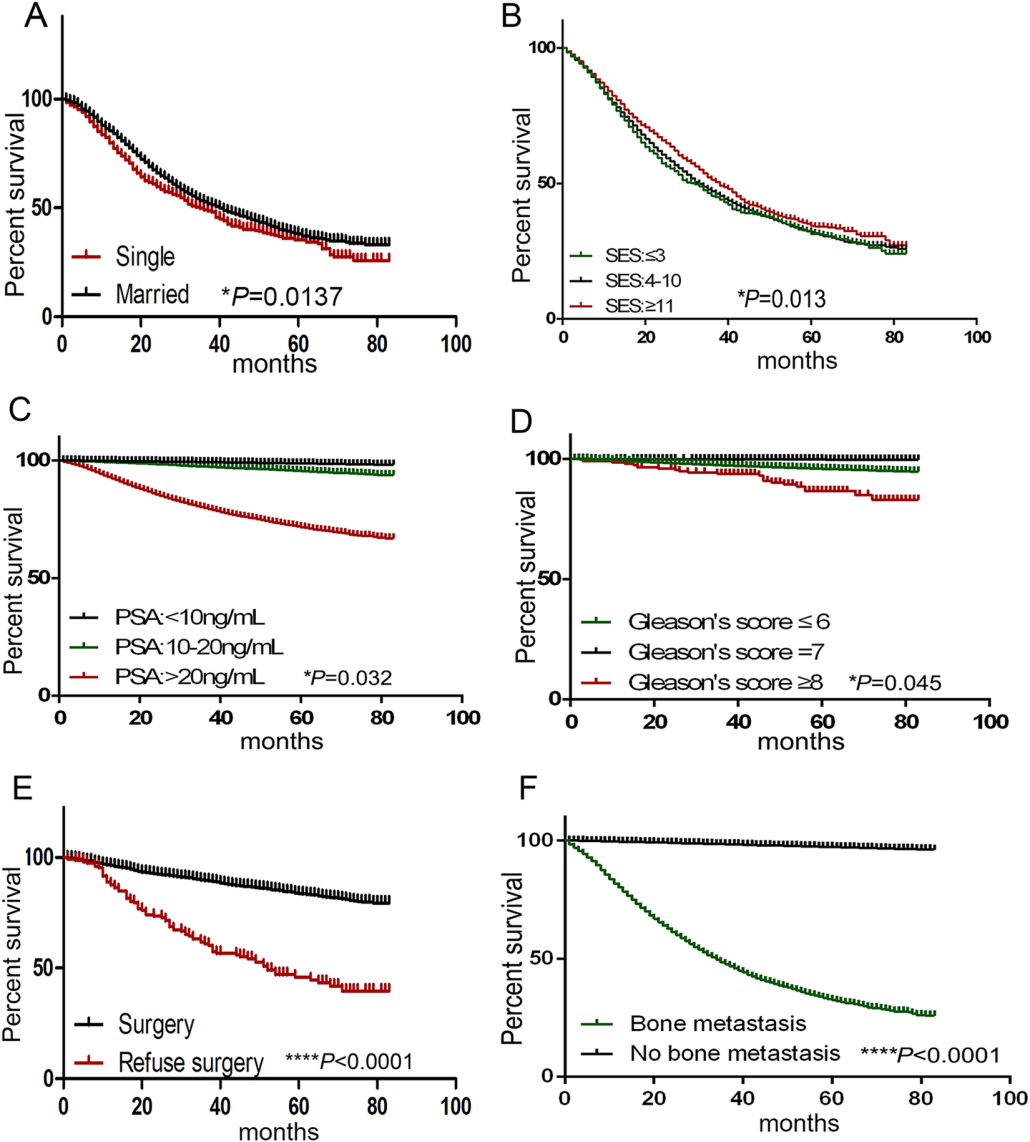
Survival curves of SEER database cohort study. (**A**) Kaplan–Meier survival curves comparing single and married marital status survival rate in patients with bone metasitasis, Log-rank test *P = 0.0137. (**B**) Kaplan–Meier survival curves showing survival rate of patients with different SES scores in SEER database cohort. Log-rank test *P = 0.013. (**C**) Kaplan–Meier survival curves shows survival rate of patients with different PSA level in this cohort, Log-rank test, *P = 0.032. (**D**) Kaplan–Meier survival curves shows survival rate of patients with different Gleason scores in this cohort. Log-rank test *P = 0.045. (**E**) Kaplan–Meier survival curves shows survival rate of surgery and refused surgery group. Patients in these two groups were with tumor stage four, Log-rank test. ****P < 0.0001. (**F**) Kaplan–Meier survival curves shows survival rate of bone metastasis and no bone metastasis patients. Log-rank test, ****P < 0.0001. Significance was determined using the log-rank test, with *P*-values < 0.05 considered statistically significant.

Married patients had a better prognosis. When we explored mortality in the ACM and CSM models, we found that compared with “single” patients, which means never-married patients, patients who are married would have lower mortality in the CSM and ACM models (HR 0.767, 95%CI 0.702–0.837, *P* < 0.001; and HR 0.662, 95% CI 0.626–0.700, *P* < 0.001, respectively). In bone metastasis patients, 3-year and 5-year survival rate in single patients were 49.08% and 35.49% which were lower than those in married patients (52.75% and 38.10%) (Fig. 1A)

Patients with a better socioeconomic status had better prognosis. Living in urban or rural places did not affect the cancer-specific mortality of patients. However, in the ACM model, patients living in rural areas have a worse outcome (HR 1.164,95% CI 1.110–1.222, *P* < 0.001). We put the lowest socioeconomic status as a reference, in the CSM and ACM models, and found that patients who have higher SES scores have a better outcome. Better socioeconomic status led to a better survival rate even in patients with bone metastasis (Fig. 1B).

A higher PSA level in patients indicated a worse outcome. After adjusting for other variates, we used a PSA level of <10 ng/mL as a reference. The higher the PSA level, the worse the overall survival (OS) is in the CSM and ACM models (Table 1). We found that in multivariate Cox regression analysis, compared with patients with a Gleason score ≤ 6, patients with a Gleason scored ≥ 8 had a worse outcome in the CSM and ACM models (Table 1). The PSA level and Gleason’s score are powerful predictors of PCa prognosis in K-M analysis (Fig. 1C,D). Our results showed that in CSM and ACM models, some patients with T1 stage might have a worse outcome than those with other stages, which might be because of the heterogeneity of PCa^24^.

Positive treatment if possible indicated a better outcome. “Surgery or not” and “radiation or not” were as variates in CSM and ACM models. Positive treatment was used as a reference, the results showed that patients with not recommendation or refused surgery would have a worse outcome (Table 1). Even in tumor stage four, the 3-year and 5-year survival rate in surgery group was 89.54% and 83.70% much more than those in refused group (61.06% and 45.95%) (Fig. 1E) Patients did not receive radiation treatment would have a worse overall survival (OS) in CSM and ACM models (Table 1).

Bone metastasis was a poor prognosis indicator. Patients with no bone metastasis would have a better OS in CSM and ACM models (HR 0.341,95%CI 0.306–0.381, *P* < 0.001; HR 0404,95%CI 0.369–0.444, *P* < 0.001). Patients with bone metastasis who are died of PCa had a 3-year and 5-year survival rate of 47.70% and 32.42%, while patients without bone metastasis had a much higher survival rate, which were 98.43% and 97.28% (Fig. 1F).

### Local patients survival analysis

We enrolled 1,335 patients with prostate cancer from 2009 to 2015 at the Second Affiliated Hospital of Zhejiang University School of Medicine. The patients characteristics are shown in Table 2 and included age, marital status, PSA level, Gleason score, TNM stage, treatment methods and bone metastasis status. A total of 98.58% of patients were 50 years old or older, which was accordance with our study cohort population from the SEER database (96.74%). Most of them were married and had a lower PSA level (<10 ng/mL). Patients with a Gleason score under 6 were in the majority. More patients in the younger group underwent surgery (68.42%) compared with those in the older group (34.65%). Furthermore, the percentage of patients in each group with a bone metastasis was higher than those in SEER database (10.53% and 8.13%).

**Table 2:** Characteristics of SEER database and local hospital data by bone metastasis status.

Character	SEER database			Local hospital data		
	Bone metastasis 6, 676 (%)	No bone metastasis 170,579 (%)	*P* value	Bone metastasis 109 (%)	No bone metastasis 1,226 (%)	*P* value
Age			<0.001			<0.001
<50	172 (2.58)	5,613 (3.29)		2 (1.83)	17 (1.39)	
50–59	1,088 (16.30)	39,644 (23.24)		13 (11.93)	206 (16.80)	
60–69	2,200 (32.95)	74,105 (43.44)		25 (22.94)	565 (46.08)	
70–79	1,789 (26.80)	41,051 (24.07)		34 (31.19)	331 (27.00)	
>80	1,427 (21.38)	10,166 (5.96)		35 (32.11)	107 (8.73)	
Race			<0.001			
White	4,929 (73.83)	127,873 (74.96)		—	—	
Black	1,297 (19.43)	26,503 (15.46)		—	—	
Asian or Pacific Islander	361 (5.41)	8,269 (4.85)		—	—	
Other	89 (1.33)	7,522 (4.41)		—	—	
Marital status^a^			<0.001			
Single	1,185 (17.75)	16,851 (9.88)		14 (12.84)	113 (9.22)	<0.001
Married	3,691 (55.29)	106,949 (62.70)		53 (48.62)	742 (60.52)	
Divorced/seprated/window/domestic partner	1,332 (19.95)	18,341 (10.75)		34 (31.19)	165 (13.46)	
Unknown	468 (7.01)	28,438 (16.67)		8 (7.34)	206 (16.80)	
Rural-urban			<0.001			
Urban	5,335 (79.91)	140,326 (82.26)		—	—	
Rural	1341 (20.09)	30,253 (17.74)		—	—	
Composite SES^b^			0.005	—	—	
≤3	828 (12.40)	18,260 (10.70)		—	—	
4–10	4,622 (69.23)	116,572 (68.34)		—	—	
≥11	1,226 (18.36)	35,747 (20.96)		—	—	
PSA (ng/mL)			<0.001			<0.001
<10	565 (8.46)	111,893 (65.60)		7 (6.42)	769 (62.72)	
10–20	549 (8.22)	21,850 (12.81)		7 (6.42)	182 (14.85)	
>20	4,851 (72.66)	12,615 (7.40)		84 (77.06)	131 (10.69)	
Unknow	711 (10.65)	24,221 (14.20)		11 (10.09)	144 (11.75)	
Gleason’s Score			<0.001			<0.001
≤6	5,042 (75.52)	164,624 (96.51)		4 (3.67)	444 (36.22)	
7	4 (0.06)	1,164 (0.68)		7 (6.42)	460 (37.52)	
≥8	8 (0.12)	198 (0.12)		52 (47.71)	240 (19.58)	
Unknown	1622 (24.30)	4597 (2.69)		46 (42.20)	82 (6.69)	
Grade^c^			<0.001			
G1	12 (0.18)	2,870 (1.68)		—	—	
G2	212 (3.18)	68,361 (40.08)		—	—	
G3	4,834 (72.41)	91,848 (53.84)		—	—	
G4	67 (1.00)	298 (0.17)		—	—	
Unknown	1,551 (23.23)	7,202 (4.22)		—	—	
Stage			<0.001	—	—	
I	0	157 (0.09)		—	—	
II	0	140,042 (82.10)		—	—	
III	0	13,908 (8.15)		—	—	
IV	6,676 (100)	5,505 (3.23)		—	—	
Unknown	0	10,967 (6.43)		—	—	
T stage			<0.001			<0.001
T1	1,792 (26.84)	68,109 (39.93)		24 (22.02)	487 (37.28)	
T2	1,993 (29.85)	78,254 (45.88)		29 (26.61)	531 (43.31)	
T3	669 (10.02)	17,131 (10.04)		13 (11.93)	140 (11.42)	
T4	784 (11.74)	876 (0.51)		7 (6.42)	9 (0.73)	
Unknown	1,438 (21.54)	6,209 (3.64)		36 (33.03)	89 (7.26)	
N stage			<0.001			<0.001
N0	3,725 (55.80)	157,451 (92.30)		56 (51.38)	1,078 (87.93)	
N1	1,557 (23.32)	3,162 (1.85)		19 (17.43)	25 (2.04)	
N2	1,394 (20.88)	9,966 (5.84)		34 (31.19)	123 (10.03)	
Surgery			<0.001			<0.001
Yes	764 (11.44)	71,210 (41.75)		11 (10.09)	458 (37.36)	
Not-Recommended	5,693 (85.28)	88,224 (51.72)		—	—	
No	219 (3.28)	11,145 (6.53)		98 (89.91)	768 (62.64)	
Radiation			<0.001			0.001
Yes	1,663 (24.91)	54,871 (32.17)		19 (17.43)	407 (33.2)	
No	5,013 (75.09)	115,708 (67.83)		90 (82.57)	819 (66.80)	

^a^Divorced includes separated; Single includes unmarried and widowed.

^b^Composite socioeconomic status.

^c^G1: well differentiated; G2: moderately differentiated; G3: poorly differentiated; G4: undifferentiated; NOS: not otherwise specified; SEER: Surveillance, Epidemiology, and End Results.

**Figure 2:**
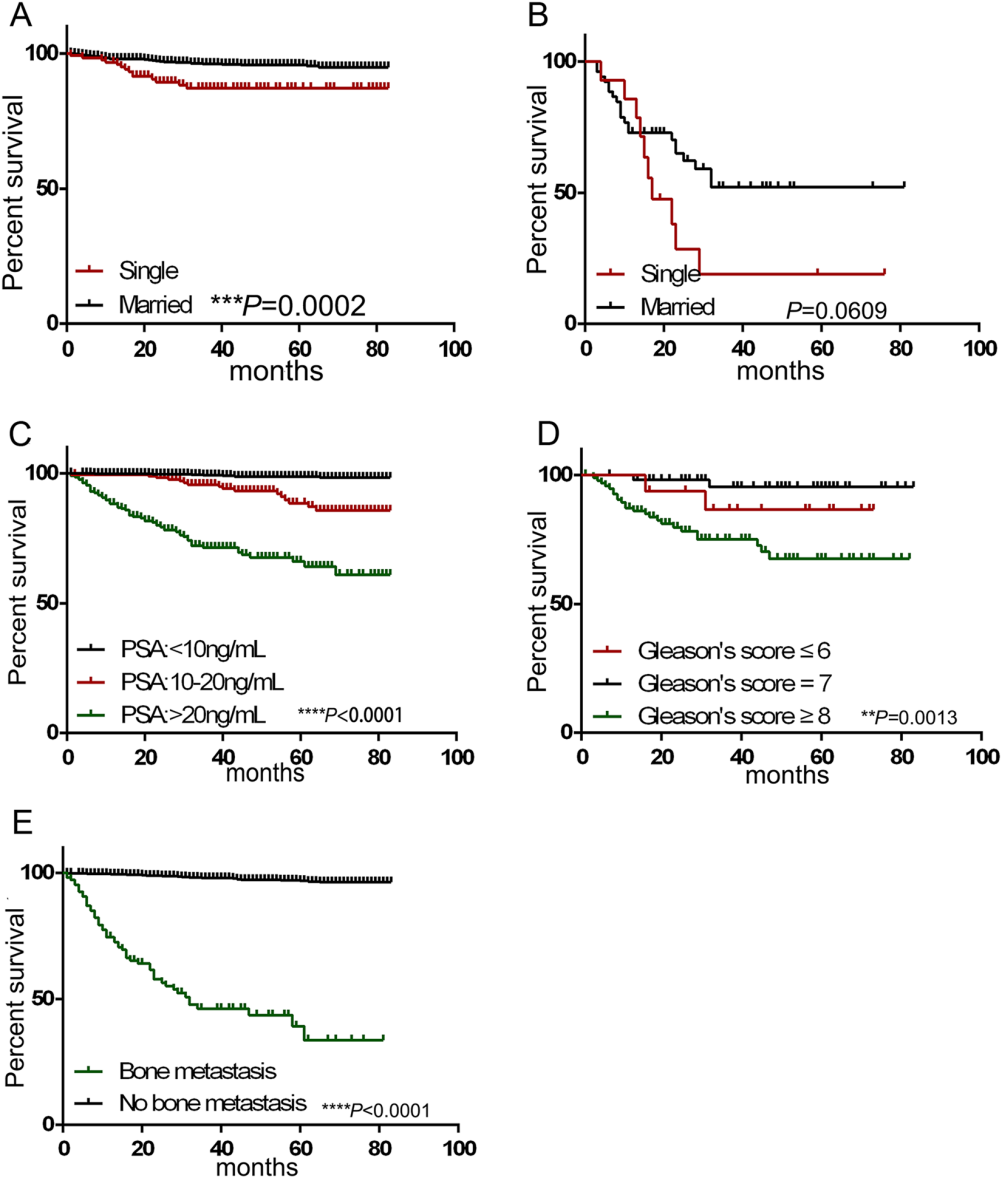
Survival curves of verified cohort in local hospital data. (**A**) Kaplan–Meier survival curves shows survival rate of “Single patients” and “Married patients”, Log-rank test. ***P = 0.0002. (**B**) Kaplan–Meier survival curves comparing single and married marital status survival rate in patients with bone metasitasis, Log-rank test, ****P < 0.0001. (**C**) Kaplan–Meier survival curves comparing survival rate of patients with different PSA levels in this cohort. Log-rank test **** *P* < 0.0001. (**D**) Kaplan–Meier survival curves showing survival rate of patients with different Gleason scores in this cohort. Log-rank test **P = 0.0013. (**E**) Kaplan–Meier survival curves comparing bone metastasis and no bone metastasis in cancer specific mortality, Log-rank test ****P < 0.0001. Significance was determined using the log-rank test, with *P*-values < 0.05 considered statistically significant.

The results showed that the 3-year and 5-year survival rates of the single group were 87.14% and 87.05%, while the rates in married group were 96.22% and 95.78%, respectively (Log-rank test, *P* = 0.0002) (Fig. 2A). For bone metastasis patients, the 3-year survival rates of the single group and married groups were 19.05% and 52.12%, respectively (Fig. 2B).

Moreover, patients with a higher PSA level had a lower OS (Log-rank test, *P* < 0.0001) (Fig. 2C), which was consistent with the results of the SEER database (Fig. 1C). Interestingly, in this cohort, patients with a Gleason score higher than 8 had a lower survival rate, and patients with a Gleason score of 7 had a higher survival rate (Fig. 2D), These results were consistent with those from the SEER database (Fig. 1D).

### Bone metastasis pattern of prostate cancer patients

In SEER database cohort, we found that bone metastasis would increase mortality in PCa. In local hospital data, the 3-year and 5-year survival rate of patients with bone metastasis were 46.15% and 39.23%, which was much lower than that of patients without bone metastasis (98.04% and 96.87%) (Fig. 2E). We categorized the characteristics of this cohort by bone metastasis, and the highest percent of bone metastasis occurred in the 60–69 age group (Table 2). As shown in Table 2, we found that approximately 26.84% and 22.02% of T1 stage patients in SEER cohort and local hospital data had bone metastasis, and 55.80% and 51.38% of N0 stage patients in these two cohort had bone metastasis, which leads to a poor prognosis. Patients with aggressive disease, such as a lower differentiated grade, also have a worse prognosis.

Multivariate logistic regression analysis was used to evaluate the relationship between the metastasis and patients characteristics by SEER database and local hospital data in Table 3 and Table S3. We found that in SEER database and local hospital data older age did not indicate a higher possibility of bone metastasis. However, a higher PSA level and Gleason score indicate a higher OR for bone metastasis (Tables 3 & S3). In SEER database cohort, compared with single patients, married patients may have a lower OR for bone metastasis (OR 0.725, 95% CI 0.604–0.872, *P* = 0.001). Patients who lived in rural areas have a higher OR for bone metastasis (*P* = 0.049).

**Table 3:** Multivariate logistic regression analysis on bone metastasis from SEER database.

Character	SEER database	
	Odds Ratio (95%CI)	*P* value
**Age**		
<50	ref.	
50–59	0.833 (0.564-1.231)	0.361
60–69	0.870 (0.595-1.272)	0.473
70–79	0.866 (0.586-1.278)	0.469
>80	1.210 (0.796-1.840)	0.371
**Race**		
White	ref.	
Black	0.925 (0.777-1.100)	0.377
Asian or Pacific Islander	0.859 (0.643-1.149)	0.307
**Marital status**^**a**^		
Single	ref.	
Married	0.725 (0.604-0.872)	0.001
Divorced/seprated/window/domestic partner	0.829 (0.664-1.036)	0.100
Rural-urban		
Urban	ref.	
Rural	1.186 (1.000-1.405)	0.049
**Composite SES**^**b**^		
≤3	ref.	
4-10	0.836 (0.680-1.027)	0.089
≥11	0.879 (0.687-1.125)	0.307
**PSA** (**ng/mL)**		
<10	ref.	
10-20	1.319 (1.069-1.627)	0.010
>20	3.793 (3.209-4.481)	<0.001
**Gleason Score**		
≤6	ref.	
7	0.932 (0.204-4.256)	0.928
≥8	2.567 (0.713-9.246)	0.149
**Grade**^**c**^		
G1	ref.	
G2	1.568 (0.234-10.523)	0.643
G3	3.028 (0.459-19.937)	0.249
G4	3.062 (0.415-22.594)	0.273
**T stage**		
T1	ref.	
T2	0.813 (0.678-0.974)	0.025
T3	0.183 (0.152-0.220)	<0.001
T4	0.272 (0.222-0.333)	<0.001
**N stage**		
N0	ref.	
N1	0.181 (0.157-0.207)	<0.001
**Surgery**		
Yes	ref.	
Not-Recommended	7.165 (6.144-8.356)	<0.001
Refused	4.462 (3.061-6.505)	<0.001
**Radiation**		
Yes	ref.	
No	1.757 (1.522-2.029)	<0.001

^a^Divorced includes separated; Single includes unmarried and widowed.

^b^Composite socioeconomic status.

^c^G1: well differentiated; G2: moderately differentiated; G3: poorly differentiated; G4: undifferentiated; NOS: not otherwise specified; SEER: Surveillance, Epidemiology, and End Results.

## Discussion

Social and clinical tumor characteristics would both effect the OS of PCa. In the current study, which were combined with SEER database and Chinese local hospital data, we found that Asian and Pacific Islander patients would achieve a better OS, which was according with the previous studies^25,26^. Married marital status was a better prognosis factor in PCa. There was no significant differences between single status and divorced status. Previous studies have shown that married patients have more knowledge of prostate cancer and that wives in homes would have nursing skills involving taking care of patients^27–30^. Although association between marital status and survival has been formerly reported in PCa patients^6–8^. We verified this conclusion with Chinese local patients.

Furthermore, the composite SES is shown in the tables, consisted of the patients’ education level, family income level, and poverty level. A higher score indicates a better socioeconomic status. We used the lowest level as a reference. The results showed that compared with the lowest level, patients with a higher SES have a better outcome in the CSM and ACM models (Table 1). Some studies have reported the importance of the relationship between prognosis and SES^21,31,32^. In PCa, some researchers showed the effect of SES and racial density on treatment and survival rate^20,33^. Some researchers focused on SES and tumor grade, which showed no positive relationship^34^. They separated SES into several parts, including self-reported education, employment status, annual household income, and neighborhood SES. However, we took the socioeconomic status as a whole, which is more complete.

When considering about the clinical indicator of prognosis, PSA level and Gleason scores can not be ignored. PSA is the most common oncological marker used for prostate cancer screening. High levels of PSA in benign prostatic hyperplasia and prostatitis decrease the specificity of PSA as a cancer marker^35^. A previous study reported that ultra-low PSA level would decreased prostate-specific survival^36^. However, in PCa patients, the PSA level is an independent prognostic factor. We confirmed this result by Chinese local hospital data. Patients with a Gleason score higher than 8 have a poor prognosis according to the CSM and ACM models (Table 1). Positive treatment if possible is a better prognosis factor even when the patients with stage four tumor progress (Fig. 1E). In SEER database we obtained this result, because of the not sufficient information of local hospital data, further research was needed.

The bone is the third most common site of metastasis in a wide range of solid tumors, including breast cancer, lung cancer, colorectal cancer, prostate cancer, gynecologic cancers, thyroid cancer, and melanoma^37^. When we checked the OR for bone metastasis, we found that compared with single patients, married patients had a lower OR (0.725, 95%CI 0.604–0.872, *P* = 0.001). Additionally, in multivariate logistic regression analysis on bone metastasis based on SEER database, a higher level of PSA had a higher OR for bone metastasis (Table 3). A higher Gleason score did not indicate a higher OR for bone metastasis (Table 3). These results suggested that we need to explore more potential molecular mechanisms of bone metastasis. Remarkbely, a low percentage of patients with T1 and N0 stage had worse prognosis due to bone metastasis. Metastasis is combined with a multi-step process during which cancer cells, responding to stimuli, detach from the primary tumor, invade the contiguous stroma, migrate over a long distance, and colonize other organs^38–40^. More investigation is needed to look for biomarkers in this group of patients.

Through the research of SEER database and the comparison and verification of local hospital data, our study provides a comprehensive prognostic factors based on social and clinical characteristics. At the same time, the conclusions of the published articles were verified and discussed. For clinicians, they could make a preliminary judgment of prognosis and metastasis by understanding the basic information of patients and clinical manifestations. For researchers, our study provides potential research directions. For example, the mechanism of T1 and N0 patients with bone metastasis, the living habits of single and married patients and the influencing factors of hormone level, and potential effect of tumor heterogeneity on PSA level.

Nevertheless, there were some limitations to our study. First, we did not analyze the influence of surgical methods on the prognosis of patients. Some surgery methods could be found in the SEER database, such as local tumor destruction or excision, subtotal or simple prostatectomy, less than total prostatectomy, and radical prostatectomy. This point is worthy of deeper exploration. Second, we did not analyze the Gleason score with 3 + 4 or 4 + 3 groups^41,42^, which would have different effects on prognosis. Third, because of the difficulty in collecting SES levels, rural or urban places and grade stage in the tissues of local patients, we did not verify these effects on prognosis.

In conclusion, our study provided prognostic factors for prostate cancer and detected a bone metastasis pattern in patients. Younger age, married marital status and Asian race were better prognosis factors in social charecteristics. Lower PSA level, lower Gleason scores, positive treatment and no bone metasitasis were better prognosis factors in clinical charecters. Additionally, according to SEER database, patients with single or divorced marital status, living in rural places, with higher PSA levels, and T1 and N0 stages have a higher OR for bone metastasis.

## Supplementary information

Supplementary information.
