# Supplementary material for: Prognosis of prostate cancer and bone metastasis pattern of patients: a SEER-based study and a local hospital based study from China
Source: Sci Rep. 2020 Jun 4;10:9104. doi: 10.1038/s41598-020-64073-6 (PMC7272631; doi:10.1038/s41598-020-64073-6)
Supplement: Supplementary file 1 — Supplementary information. [file 41598_2020_64073_MOESM1_ESM.docx]

Prognosis of prostate cancer and bone metastasis pattern of patients: a SEER-based study and a local hospital based study from China

Dongyu Liu^1#^, Yue Kuai^2#^, Ruohui Zhu^3^, Chenhe Zhou^1^, Yiqing Tao^1^, Weidong Han^2^*, Qixin Chen^1^*

^1^ Department of Orthopedics Surgery, 2nd Affiliated Hospital, School of Medicine, Zhejiang University,Hangzhou,China ; ^2^Department of Medical Oncology, Sir Run Run Shaw Hospital, College of Medicine, Zhejiang University, Hangzhou, Zhejiang, China;^3^ Department of Internal Medicine, Lincoln Medical Center,234 E149th Street, The Bronx, NY 10451, USA;

Email:

Dongyu Liu: dongyvliu@163.com

Yue Kuai: kuaiyue@zju.edu.cn

Ruohui Zhu: ruohuizhu123@gmail.com

Chenhe Zhou: andyzhou@zju.edu.cn

Yiqing Tao:taoyq@zju.edu.cn

# Equal contributors

*Corresponding Author: Weidong Han, Sir Run Run Shaw Hospital, School of Medicine, Zhejiang University, 3# East Qinchun Road, Hangzhou310016, Zhejiang, China,. Tel: +86-571-86006926; E-mail: [hanwd@zju.edu.cn](mailto:hanwd@zju.edu.cn); Qixin Chen, M.D., Ph.D., Department of Orthopedics Surgery, 2nd Affiliated Hospital, School of Medicine, Zhejiang University, 88# Jiefang Road, Hangzhou 310009, Zhejiang, China; E-mail: [zrcqx@zju.edu.cn](mailto:zrcqx@zju.edu.cn) Tel: +86-571 8778 3543; Fax: +86-571 8778 3543

Table S1. Characteristics of SEER database included cohort by age.

| Character | Age :18-49  N=5785(%) | Age :≥50  N=171,470(%) | *P* value |
| --- | --- | --- | --- |
| Race |  |  | <0.001 |
| White | 3,706 (64.06) | 129,096(75.29) |  |
| Black | 1,709(29.54) | 26,503(15.46) |  |
| Asian or Pacific Islander | 175(3.03) | 8,455(4.93) |  |
| Other | 195(3.37) | 7,416(4.32) |  |
| Marital status^a^ |  |  | <0.001 |
| Single | 1,060(18.32) | 16,976(9.90) |  |
| Married | 3,445(59.55) | 107,195(65.52) |  |
| Divorced/seprated/window/  domestic partner | 460(7.95) | 19,213(11.20) |  |
| Unknown | 820(14.17) | 28,086(16.38) |  |
| Rural-urban |  |  | <0.001 |
| Urban | 5060(87.47) | 140,601(82) |  |
| Rural | 725(12.53) | 30,869(18) |  |
| Composite SES^b^ |  |  | 0.005 |
| ≤3 | 550(9.51) | 18,538(10.81) |  |
| 4-10 | 3,988(68.94) | 117,206(68.35) |  |
| ≥11 | 1,247(21.56) | 35,726(20.84) |  |
| PSA(ng/mL) |  |  | <0.001 |
| <10 | 4,266(73.74) | 108,192(63.10) |  |
| 10-20 | 480(8.30) | 21,919(12.78) |  |
| >20 | 437(7.55) | 17,029(9.93) |  |
| Unknow | 602(10.41) | 24,330(14.19) |  |
| Gleason Score |  |  | 0.001 |
| ≤6 | 5,557(96.06) | 164,109(95.71) |  |
| 7 | 57(0.99) | 1,107(0.65) |  |
| ≥8 | 6(0.10) | 200(0.12) |  |
| Unknown | 165(2.85) | 6,054(3.53) |  |
| Grade^c^ |  |  | <0.001 |
| G1 | 109(1.88) | 2,773(1.63) |  |
| G2 | 2,769(47.87) | 65,804(38.38) |  |
| G3 | 2,638(45.60) | 94,044(54.85) |  |
| G4 | 10(0.10) | 355(0.21) |  |
| Unknown | 257(4.48) | 8,494(4.94) |  |
| Stage |  |  | <0.001 |
| I | 0 | 157(0.09) |  |
| II | 4,772(82.49) | 135,270(78.89) |  |
| III | 447(7.73) | 13,461(7.85) |  |
| IV | 367(6.34) | 11,814(6.89) |  |
| Unknown | 199(3.44) | 10,768(6.28) |  |
| T stage |  |  | <0.001 |
| T1 | 1,435(24.81) | 68,466(39.93) |  |
| T2 | 3,563(61.59) | 76,684(44.72) |  |
| T3 | 599(10.35) | 17,201(10.03) |  |
| T4 | 50(0.86) | 1,610(0.94) |  |
| Unknown | 138(2.39) | 7,509(4.38) |  |
| N stage |  |  | <0.001 |
| N0 | 5,389(93.15) | 155,787(90.85) |  |
| N1 | 172(2.97) | 4,547(2.65) |  |
| N2 | 224(3.87) | 11,136(6.49) |  |
| Surgery |  |  | <0.0001 |
| Yes | 3,683(63.66) | 68,291(39.83) |  |
| Not-Recommended | 1,816(31.39) | 92,101(53.71) |  |
| Refused | 286 (4.94) | 11,078(6.46) |  |
| Radiation |  |  | <0.001 |
| Yes | 1,029(17.79) | 55,505(32.37) |  |
| No | 4,756(82.21) | 115,965(67.63) |  |
| Bone Metastasis |  |  | 0.001 |
| Yes | 172(2.97) | 6,504(3.79) |  |
| No | 5,613(97.03) | 164,966(96.21) |  |
|  |  |  |  |

^a^ Divorced includes separated; Single includes unmarried and widowed

^b^ Composite socioeconomic status

^c^ G1: well differentiated; G2: moderately differentiated; G3: poorly
differentiated; G4: undifferentiated; NOS: not otherwise specified; SEER: Surveillance, Epidemiology, and End Results.

TableS2. Characteristics of local hospital included cohort by age

| Character | Age 18-49  N=19(%) | Age≥50  N=1,316(%) | *P* value |
| --- | --- | --- | --- |
| Marital status^a^ |  |  | 0.003 |
| Single | 6 (31.58) | 121 (9.19) |  |
| Married | 6 (31.58) | 789 (59.95) |  |
| Divorced/seprated/window/  domestic partner | 2 (10.53) | 197 (14.97) |  |
| Unknown | 5 (26.32) | 209 (15.88) |  |
| PSA(ng/mL) |  |  | 0.335 |
| <10 | 13 (68.42) | 763 (57.98) |  |
| 10-20 | 0 | 189 (14.36) |  |
| >20 | 4 (21.05) | 211 (16.03) |  |
| Unknow | 2 (10.53) | 153 (11.63) |  |
| Gleason’s Score |  |  | 0.301 |
| ≤6 | 10 (52.63) | 438 (33.28) |  |
| 7 | 5 (26.32) | 462 (35.11) |  |
| ≥8 | 2 (10.53) | 290 (22.04) |  |
| Unknown | 2 (10.53) | 126 (9.57) |  |
| T stage |  |  | 0.425 |
| T1 | 4 (21.05) | 477 (36.25) |  |
| T2 | 12 (63.16) | 548 (41.64) |  |
| T3 | 2 (10.53) | 151 (11.47) |  |
| T4 | 0 | 16 (1.22) |  |
| Unknown | 1 (5.26) | 124 (9.42) |  |
| N stage |  |  | 0.258 |
| N0 | 18 (94.74) | 1,116 (84.80) |  |
| N1 | 1(5.26) | 43 (3.27) |  |
| NX | 0 | 157 (11.93) |  |
| Surgery |  |  | 0.002 |
| Yes | 13 (68.42) | 456 (34.65) |  |
| No | 6 (31.58) | 860(65.35) |  |
| Radiation |  |  |  |
| Yes | 4 (21.05) | 422 (32.07) | 0.306 |
| No | 15 (78.95) | 894 (67.93) |  |
| Bone Metastasis |  |  |  |
| Yes | 2 (10.53) | 107 (8.13) | 0.705 |
| No | 17 (89.47) | 1,209 (91.87) |  |
|  |  |  |  |
|  |  |  |  |
|  |  |  |  |
|  |  |  |  |
|  |  |  |  |
|  |  |  |  |
|  |  |  |  |
|  |  |  |  |
|  |  |  |  |
|  |  |  |  |
|  |  |  |  |
|  |  |  |  |
|  |  |  |  |
|  |  |  |  |
|  |  |  |  |

^a^ Divorced includes separated; Single includes unmarried and widowed

^b^ Composite socioeconomic status

^c^ G1: well differentiated; G2: moderately differentiated; G3: poorly
differentiated; G4: undifferentiated; NOS: not otherwise specified; SEER: Surveillance, Epidemiology, and End Results.

Table S3. Multivariate logistic regression analysis on bone metastasis from local hospital data

|  | Local hospital data | |
| --- | --- | --- |
| Character | Odds Ratio(95%CI) | *P* value |
| Age |  |  |
| <50 | Empty |  |
| 50-59 | 2.860(0.660-12.388) | 0.160 |
| 60-69 | 1.472(0.416-5.209) | 0.549 |
| 70-79 | 1.603(0.498-5.161) | 0.429 |
| >80 | Omitted |  |
| Marital status^a^ |  |  |
| Single | Ref. |  |
| Married | 1.450(0.425-4.952) | 0.553 |
| Divorced/seprated/window/  domestic partner | 2.231(0.574-8.680) | 0.247 |
| PSA(ng/mL) |  |  |
| <10 | Ref. |  |
| 10-20 | 3.664(0.840-15.996) | 0.084 |
| >20 | 28.108(8.414-93.900) | <0.001 |
| Gleason Score |  |  |
| ≤6 |  |  |
| 7 | 1.286(0.282-5.865) | 0.745 |
| ≥8 | 7.927(1.894-33.182) | 0.005 |
| T stage |  |  |
| T1 | Ref. |  |
| T2 | 1.184(0.479-2.922) | 0.982 |
| T3 | 1.129(0.300-4.248) | 0.857 |
| T4 | 0.777(0.091-6.624) | 0.818 |
| N stage |  |  |
| N0 | Ref |  |
| N1 | 1.356(0.338-5.443) | 0.43 |
| Surgery |  |  |
| Yes | Ref |  |
| No | 5.845(1.787-19.130) | 0.003 |
| Radiation |  |  |
| Yes | Ref. |  |
| No | 6.074(2.401-15.363) | <0.001 |
|  |  |  |

^a^ Divorced includes separated; Single includes unmarried and widowed

SEER: Surveillance, Epidemiology, and End Results.
